# Metal–Support Interactions and C1 Chemistry: Transforming Pt-CeO_2_ into a Highly Active and Stable Catalyst for the Conversion of Carbon Dioxide and Methane

**DOI:** 10.1021/acscatal.0c04694

**Published:** 2021-01-20

**Authors:** Feng Zhang, Ramón A. Gutiérrez, Pablo G. Lustemberg, Zongyuan Liu, Ning Rui, Tianpin Wu, Pedro J. Ramírez, Wenqian Xu, Hicham Idriss, M. Verónica Ganduglia-Pirovano, Sanjaya D. Senanayake, José A. Rodriguez

**Affiliations:** † Department of Materials Science and Chemical Engineering, SUNY at Stony Brook, Stony Brook, New York 11794, United States; ‡ Facultad de Ciencias, Universidad Central de Venezuela, Caracas 1020-A, Venezuela; § Instituto de Física Rosario (IFIR), CONICET-UNR, Bv. 27 de Febrero 210bis, Rosario, Santa Fe S2000EZP, Argentina; ∥ Instituto de Catálisis y Petroleoquímica, CSIC, C/Marie Curie 2, Madrid 28049, Spain; ⊥ Chemistry Division, Brookhaven National Laboratory, Upton, New York 11973, United States; # X-ray Science Division, Advanced Photon Source, Argonne National Laboratory, Argonne, Illinois 60439, United States; ∇ Zoneca-CENEX, R&D Laboratories, Alta Vista, Monterrey 64770, México; ○ SABIC Corporate Research & Development (CRD), KAUST, Thuwal 29355, Saudi Arabia

**Keywords:** C1 chemistry, CO_2_ conversion, CH_4_ conversion, methane dry reforming, platinum, metal−support interactions

## Abstract

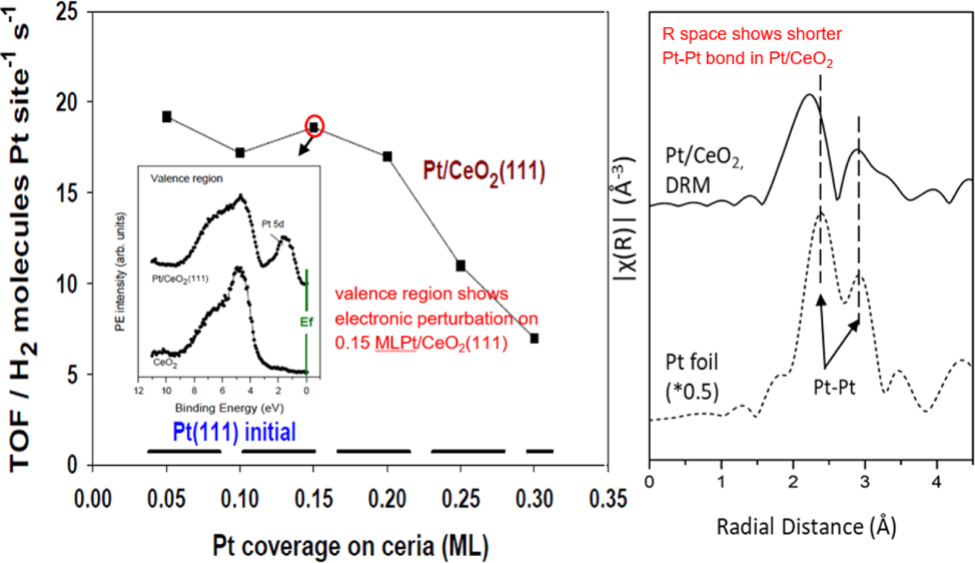

There is an ongoing search for materials which can accomplish the activation of two dangerous greenhouse gases like carbon dioxide and methane. In the area of C1 chemistry, the reaction between CO_2_ and CH_4_ to produce syngas (CO/H_2_), known as methane dry reforming (MDR), is attracting a lot of interest due to its green nature. On Pt(111), high temperatures must be used to activate the reactants, leading to a substantial deposition of carbon which makes this metal surface useless for the MDR process. In this study, we show that strong metal–support interactions present in Pt/CeO_2_(111) and Pt/CeO_2_ powders lead to systems which can bind CO_2_ and CH_4_ well at room temperature and are excellent and stable catalysts for the MDR process at moderate temperature (500 °C). The behavior of these systems was studied using a combination of in situ/*operando* methods (AP-XPS, XRD, and XAFS) which pointed to an active Pt-CeO_2-*x*_ interface. In this interface, the oxide is far from being a passive spectator. It modifies the chemical properties of Pt, facilitating improved methane dissociation, and is directly involved in the adsorption and dissociation of CO_2_ making the MDR catalytic cycle possible. A comparison of the benefits gained by the use of an effective metal-oxide interface and those obtained by plain bimetallic bonding indicates that the former is much more important when optimizing the C1 chemistry associated with CO_2_ and CH_4_ conversion. The presence of elements with a different chemical nature at the metal-oxide interface opens the possibility for truly cooperative interactions in the activation of C–O and C–H bonds.

## Introduction

Carbon dioxide (CO_2_) is a common greenhouse gas emitted whenever coal, oil, or other carbon-rich fuels are burned. It is the largest contributor to climate change.^1^ The conversion of CO_2_ to high value chemicals or fuels is an important topic which is attracting a lot of attention worldwide. In nature, the methane molecule (CH_4_) is highly abundant, being the simplest and most stable alkane compound. While methane does not linger as long in the atmosphere as carbon dioxide, it is far more devastating to the climate because of how effectively it absorbs heat.^3−5^ CH_4_ has a greenhouse warming potential (GWP) which is 84 times greater than that of CO_2_. It has been estimated that methane can be responsible for 25% of already observed changes to Earth’s climate.^3−5^ CH_4_ is the main component of natural gas and is frequently flared or vented into the atmosphere during oil and gas drilling operations. As in the case of CO_2_, there are important environmental and commercial interests to activate and transform CH_4_ into value-added chemicals (olefins, aromatics, and alcohols).^2,4,5^

In the area of C1 chemistry, the reaction between CO_2_ and CH_4_ to produce syngas (CO/H_2_), methane dry reforming (MDR), has attracted a lot of interest due to its green nature.^6^ The syngas produced by this reaction can be used in fuel cells fed with H_2_, in the synthesis of methanol or other oxygenates, and in the production of hydrocarbons through the Fischer-Tropsch process.^6^ Two main reactions in the MDR process involve the conversion of CO_2_:
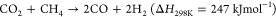
1
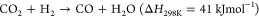
2

In the second reaction, the reverse water-gas shift (RWGS), is often seen at high temperature. The MDR process is a real challenge due to the high stability and the nonpolar nature of both CO_2_ and CH_4_.^2,7−9^ Heterogeneous catalysts are frequently used to accomplish this task and the activation of C–O and C–H bonds must be done in a concerted manner to avoid carbon deposition and subsequent deactivation of the catalyst.^6,8^ When dealing with the activation of CO_2_ and CH_4_ on metal and oxide surfaces, a set of scaling relations and descriptors have been evaluated for the controlled cleavage of the C–O or C–H bonds in these molecules.^7−15^ However, what types of systems can simultaneously activate CO_2_ and CH_4_? It has become clear that single metals alone are not efficient for the MDR process^16,17^ and better results can be obtained when one uses metal–metal or metal-oxide interfaces where different sites cooperate in the activation of CO_2_ and CH_4_.^8,18−20^

In recent years, great research efforts have been made in order to develop metal/oxide catalysts with good activity, selectivity, and stability for the MDR process.^6,8^ Systems which contain noble metals (Rh, Ru, Pt, and Ir) have received substantial attention since they can be very active and less sensitive to deactivation by carbon deposition than catalysts based on Ni or Co.^6,21−24^ Pt and Pt alloys have been investigated showing a remarkable potential,^6,25−29^ but important issues such as the effect of the metal particle size or morphology and the role of the support (Al_2_O_3_, MgO, CeO_2_, ZrO_2_, CeO_2_-ZrO_2_, and carbon) need to be addressed for optimizing this type of MDR catalysts.^6^ In this article, we investigate the MDR process on well-defined Pt/CeO_2_(111) and powder Pt/CeO_2_ catalysts using a multitechnique approach.

On surfaces of noble metals, methane exhibits a rather low probability for dissociation which makes the effective conversion of the molecule difficult.^30−32^ For example, in the case of clean Pt(111), used as a typical benchmark substrate in fundamental studies of hydrocarbon activation, a value of ∼1 × 10^–8^ has been measured for the methane C–H dissociation probability at 25 °C.^32^ At this temperature, the hydrocarbon molecule dissociates depositing C and CH_3_ groups on the platinum surface. In the range of 100–200 °C, the formation of C–C bonds occurs yielding species such as ethylidyne (C_2_H_3_) and ethynyl (C_2_H) on the platinum surface.^32^ A carbonaceous layer eventually deactivates the chemical and catalytic properties of Pt(111).^32^ The same occurs when Pt(100) or Pt(110)-(1×2) are exposed to methane at elevated temperature.^33,34^ The platinum surfaces also show very poor activation of CO_2_.^7^ Neither Pt(111) nor Pt(100) bind carbon dioxide well.^7^ Recent works have found special electronic and chemical properties in Pt atoms directly bonded to ceria,^35−37^ but no systematic research has been carried out for the reaction of CO_2_ and CH_4_ over Pt-CeO_2_ interfaces. Can metal–support interactions be useful for MDR and in the control of carbon deposition on platinum? In this article, we show clear evidence of metal–support interactions in the Pt/CeO_2_ system and their effects in shifting the system away from the normal behavior of bulk Pt, producing active and stable catalysts for CO_2_ and CH_4_ activation in dry reforming.

## Results and Discussion

### Reaction of CH_4_ and CO_2_ on Pt/CeO_2_(111) Surfaces

**Figure 1:**
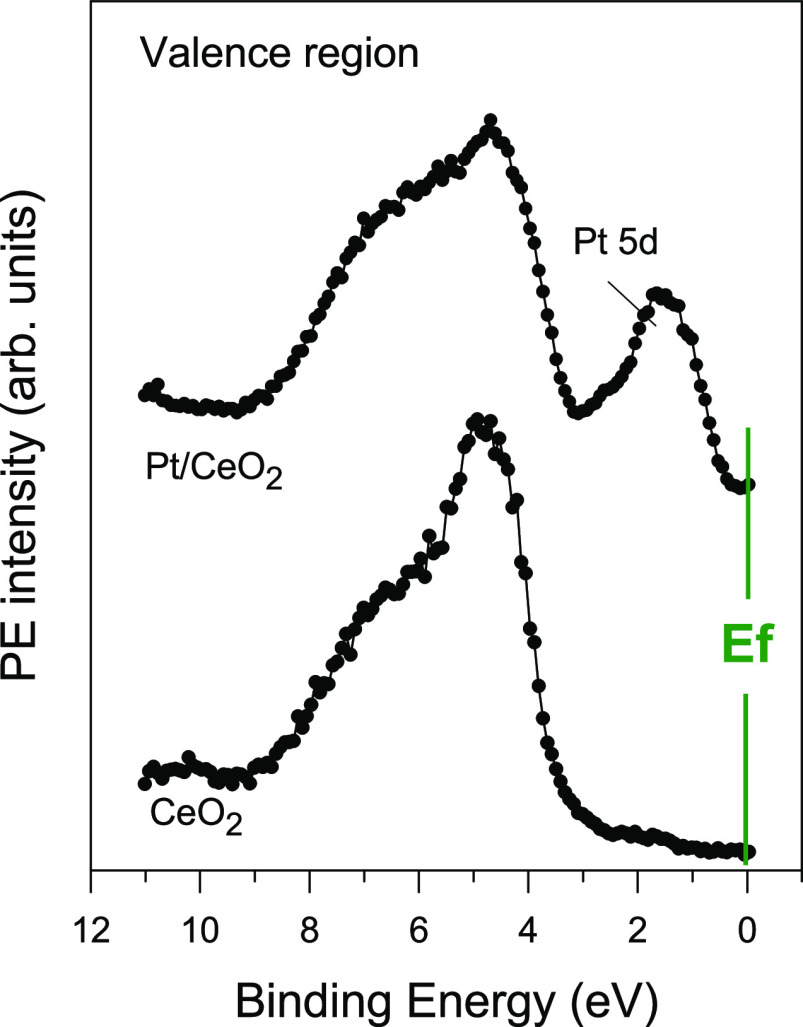
He-II valence photoelectron spectra collected before and after deposition of 0.15 ML of Pt on a CeO_2_(111) surface.

We started by investigating the interaction of CH_4_, CO_2_, and CH_4_/CO_2_ mixtures with Pt/CeO_2_(111) surfaces. Figure 1 shows valence photoemission spectra for a clean CeO_2_(111) surface and a surface containing 0.15 monolayer(ML) of platinum. The valence spectrum for the ceria system exhibits the O 2p band between 8 and 3.5 eV with a large band gap below the Fermi level (E_f_). The addition of Pt led to the appearance of new features centered at a binding energy of 2 to 1 eV. These features come from Pt 5d, 6s states. It is important to notice that the density of states (DOS) around the Fermi level for Pt/CeO_2_(111) is close to zero. This is very different from the valence photoemission spectrum of bulk platinum, Pt(111) or Pt(100), where a very large DOS is seen at the Fermi level.^37−39^ Thus, the Pt atoms in contact with ceria exhibit very strong electronic perturbations which can affect their chemical and catalytic properties. This phenomenon was seen over a wide range of temperatures (25–600 °C) but only at small coverages of Pt (< 0.2 ML). For higher coverages of the admetal (> 0.5 ML), the valence spectrum of Pt/CeO_2_(111) eventually converged to that of bulk platinum and the novel chemical behavior of the admetal disappeared.^37^ The data of photoelectron spectroscopy at low Pt coverages are consistent with previous theoretical studies which show electronic perturbations when atoms or small clusters of the metal are in contact with a ceria surface.^33−35,40,41^

**Figure 2:**
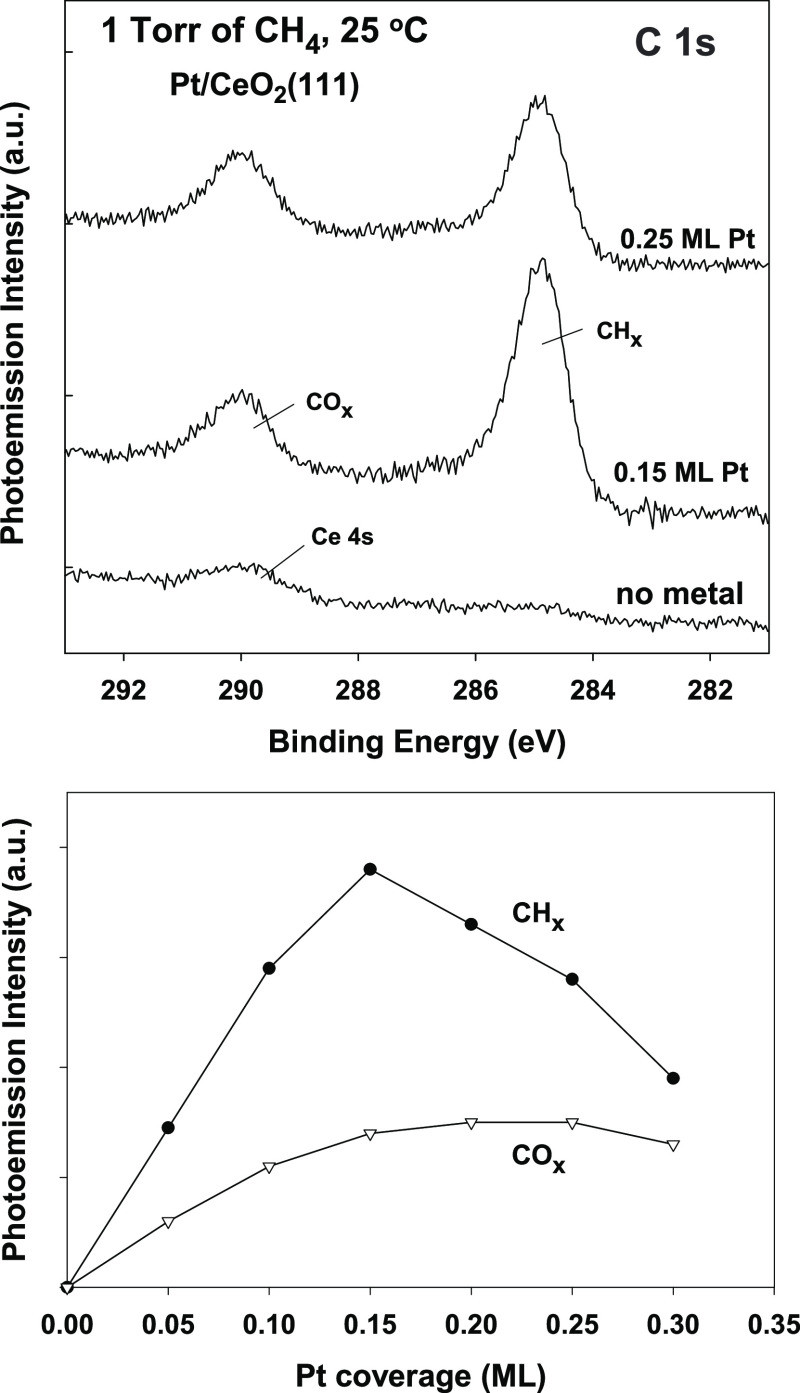
Top panel: C 1s XPS spectra collected after exposing plain CeO_2_(111) and Pt/CeO_2_(111) surfaces to 1 Torr of methane at 25 °C for 5 min. Bottom panel: Variation in the signal for CH*_x_* and CO*_x_* species in the C 1s region as a function of admetal coverage in Pt/CeO_2_(111).

Figure 2 displays C 1s X-ray photoelectron spectroscopy (XPS) spectra recorded after dosing CH_4_ at 25 °C to plain CeO_2_(111) and surfaces precovered with 0.15 and 0.25 ML of platinum. CeO_2_(111) did not dissociate the alkane molecule at 25 °C. In contrast, methane dissociation occurred in the case of Pt/CeO_2_(111). Strong features around 284.8 eV indicate the existence of CH*_x_* (*x* = 1, 2, and 3) species formed by the partial dissociation of methane {CH_4_ → CH*_x_* + (4-*x*) H} on the surface.^18−20^ A second peak located near 290 eV points to the formation of carbonate-like CO*_x_* species as a final product of the full decomposition of methane.^18−20^ The Pt/CeO_2_(111) surfaces in Figure 2 exhibit a reactivity toward methane higher than that seen for surfaces of the bulk metal such as Pt(111), Pt(100), or Pt(110)-(1×2).^31−34^

The reactivity of the Pt/CeO_2_(111) surfaces to dissociate methane at 25 °C depended strongly on the amount of platinum dispersed on ceria. The highest reactivity was seen for the system with 0.15 ML (see Figure 2). This correlates with the large electronic perturbations seen in valence photoemission (Figure 1). At higher coverages of Pt (> 0.2 ML), the electronic perturbations on Pt decreased,^37^ and the amount of CH*_x_* and CO*_x_* deposited on the surface upon exposure also dropped (bottom panel in Figure 2). Previous theoretical studies have indicated that electronic perturbations associated with Pt-CeO_2_ bonding can largely reduce the barrier for the activation of C–H bonds in methane.^42^

**Figure 3:**
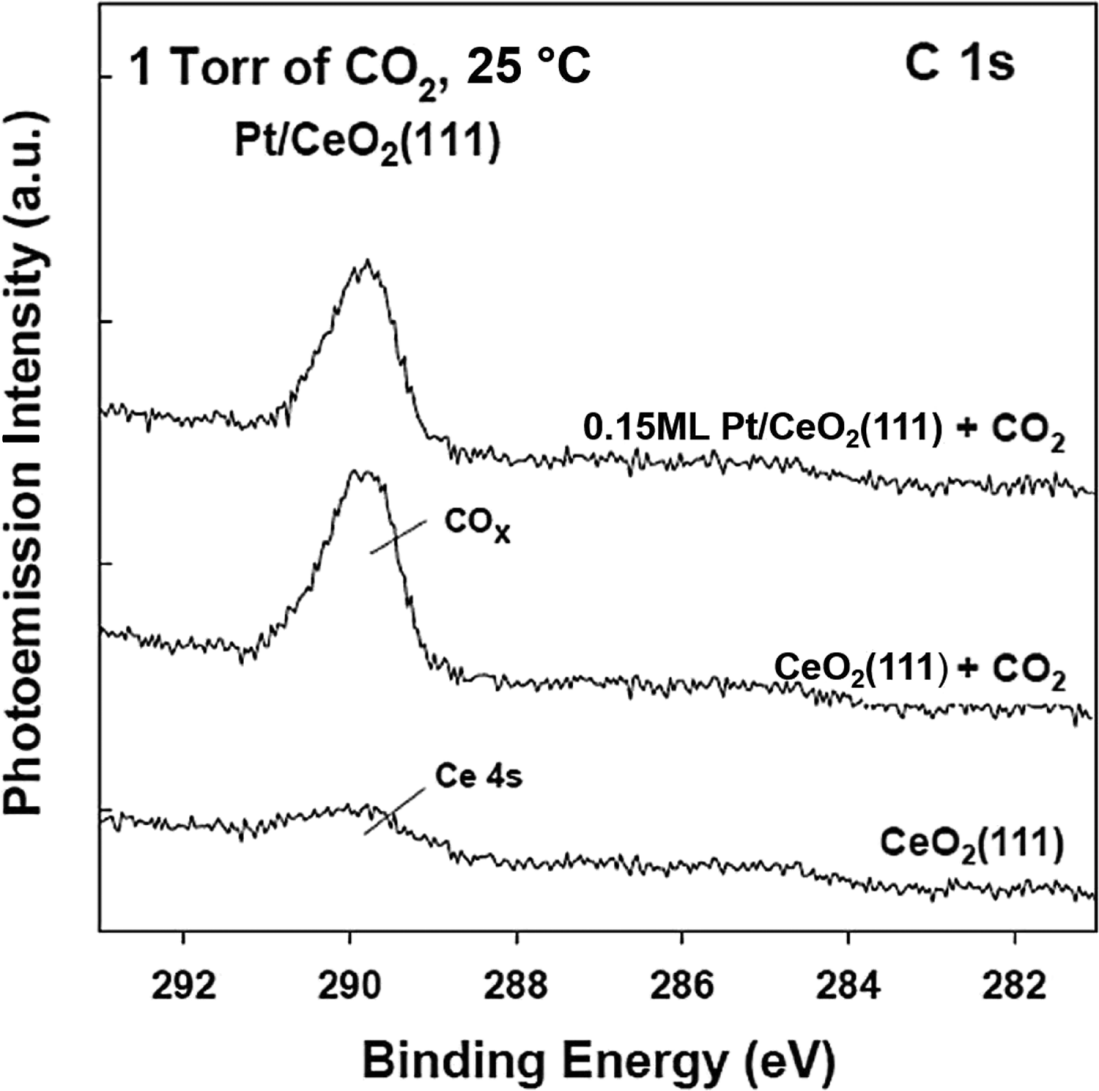
C 1s XPS spectra collected after exposing plain CeO_2_(111) and a Pt/CeO_2_(111) surface to 1 Torr of CO_2_ at 25 °C for 5 min. The carbonate (CO*_x_*) peak did not disappear when the surface was heated to temperatures as high as 500 °C.

Figure 3 displays C 1s XPS spectra acquired after dosing 1 Torr of CO_2_ to clean CeO_2_(111) and an oxide surface precovered with 0.15 ML of platinum at 25 °C. For both systems, the adsorption of CO_2_ produces a peak around 290 eV which can be assigned to a carbonate (CO*_x_*) species produced by direct reaction of CO_2_ with O sites of the surface.^18−20^ The presence of Pt did not lead to the growth of clear peaks for adsorbed CO_2_ or CO on the metal, but the occurrence of a reaction of the CO_2_(gas) → CO(gas) + O(surface) type cannot be ruled out. XPS results showed that platinum was oxidized from mainly Pt^1+^ to Pt^2+^ upon exposure to CO_2_. This is remarkable because neither Pt(111) nor Pt(100) bind carbon dioxide well.^7^ The similarities of the carbonate peaks in Figure 3 suggest that the adsorbed CO_2_ mainly interacted with the ceria support. Thus, our XPS results indicated that CH_4_ and CO_2_ can be adsorbed on Pt/CeO_2_(111) at room temperature, but they did not react to yield syngas as expected by the MDR process. Catalytic activity was observed at temperatures above 400 °C after methane reduced ceria producing O vacancies where CO_2_ dissociated.

**Figure 4:**
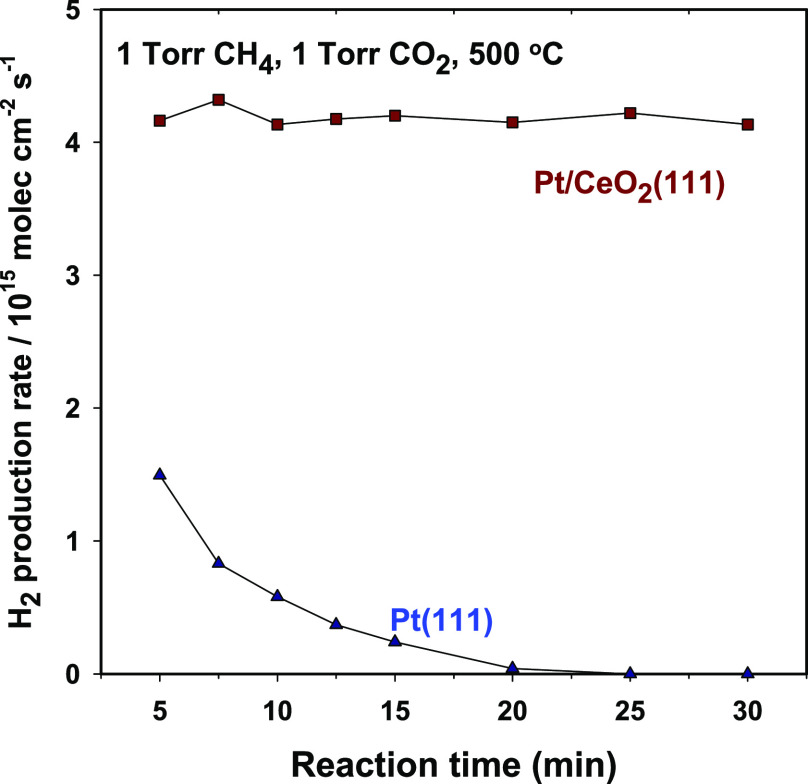
Production of H_2_ by methane dry reforming on Pt(111) and 0.15 ML of Pt supported on CeO_2_(111). Reaction conditions: 1 Torr of CH_4_, 1 Torr of CO_2_, and 500 °C.

A batch reactor was used to test the catalytic performance of plain Pt(111), CeO_2_(111), and 0.15 ML of Pt deposited on a CeO_2_(111) surface. At the reaction conditions examined (1 Torr of CH_4_, 1 Torr of CO_2_, 400–500 °C), neither Pt(111) nor CeO_2_(111) showed any sustained activity for the MDR reaction. In the case of Pt(111), some catalytic activity was initially observed but it dropped continuously and, after 20 min of reaction, no catalytic activity was seen (Figure 4). Postreaction characterization with XPS showed that the plain platinum surface was poisoned by a thick carbon layer generated by the decomposition of methane. On this system, CO_2_ could not dissociate fast enough to provide the O for the removal of the carbon generated by methane.^32^ In addition, Pt(111) is known to be active for the Boudouard reaction (2CO → C + CO_2_) which also could induce platinum deactivation by carbon poisoning.

In contrast to the behavior of Pt(111), our kinetic data shown in Figure 4 indicate that a catalyst generated by depositing 0.15 ML of Pt on a CeO_2_(111) surface is highly active and stable for the MDR reaction. The metal–support interactions in the Pt-CeO_2_ interface lead to an excellent catalytic performance. Furthermore, these interactions also substantially reduce the rate of the Boudouard reaction with respect to Pt(111).^37^ Thus, both factors make Pt/CeO_2_(111) a very good catalyst for the MDR process. Postreaction characterization with XPS gave a negligible amount of C on Pt/CeO_2_(111) after 30 min of reaction under MDR conditions. In the C1s XPS region, a peak for carbonate, similar to that seen in Figure 3, was seen. Furthermore, upon finishing the postreaction characterization with XPS, the sample was transferred back to the reactor and the MDR process was performed under the same conditions as those shown in Figure 4 for an additional 2 h, observing the same rate of H_2_ production and no measurable deposition of carbon on the surface of the catalyst.

**Figure 5:**
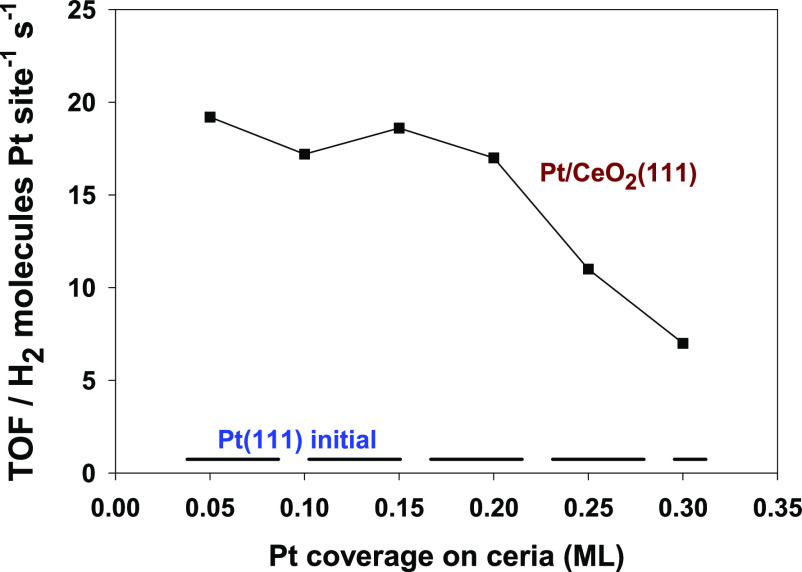
Calculated turnover frequencies for Pt/CeO_2_(111) surfaces. For comparison, the initial TOF of Pt(111), before it was deactivated by carbon deposition, is included as the dashed line at the bottom. Reaction conditions: 1 Torr of CH_4_, 1 Torr of CO_2_, and 500 °C.

Figure 5 displays the calculated turnover frequency (TOF) at 500 °C for different Pt/CeO_2_(111) systems as a function of admetal coverage. The TOFs were calculated assuming that all the Pt atoms which were present on the ceria support were active in the catalytic process. For comparison, as shown in Figure 5, we also include the initial TOF for Pt(111) before its surface was deactivated by carbon deposition. Results shown in Figure 5 indicate that at small coverages of Pt, the dispersed particles on ceria are at least 20 times more active than plain Pt(111). Thus, the remarkable increase in the activity and stability of Pt might be linked to the strong interactions of small coverages of Pt with CeO_2_. As shown in Figure 5, the TOF decreases when the Pt coverage goes above 0.2 ML—a phenomenon which correlates with a reduction in the electronic perturbations^37^ in Pt and in the reactivity of the admetal toward methane (Figure 2).

**Figure 6:**
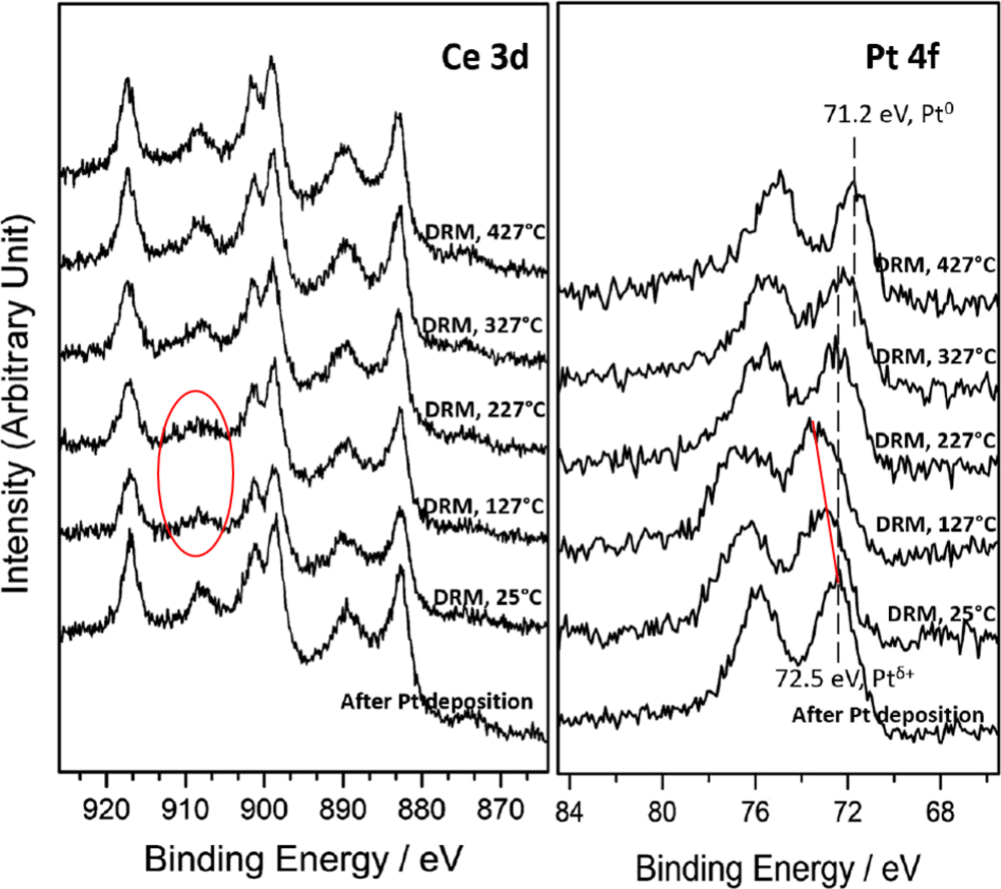
Ce 3d and Pt 4f AP-XPS spectra of Pt/CeO_2_ (111) for the MDR reaction at elevated temperatures. Spectra were collected at 25, 127, 227, 327, and 427 °C in a 50 mTorr CH_4_ + 50 mTorr CO_2_ gas atmosphere.

Ambient pressure-XPS (AP-XPS) was used to study the chemical state of a Pt/CeO_2_(111) catalyst when exposed to a reactant CH_4_/CO_2_ mixture in a large range of temperature (Figure 6). The initial position of the Pt 4f peak indicates that Pt is oxidized with a Pt^+^-dominating feature upon deposition on CeO_2_(111) at 25 °C.^40−42^ The exposure to the CH_4_ and CO_2_ gas mixture induced a peak shift of the Pt signal to higher binding energy at 25 and 127 °C. This peak shift could be attributed to the adsorbed CO*_x_*/CH*_x_* species (resulting from CH_4_ dissociation and CO_2_ binding, see Figures 2 and 3) on the Pt surface which increased the work function and binding energy.^43,44^ An analysis of the corresponding Ce 3d XPS spectra indicates that there may be some reduction of Ce^4+^ into Ce^3+^ upon exposure to the CH_4_/CO_2_ gas mixtures at different temperatures. As can be seen, upon deposition of Pt, most of the ceria is in the 4+ state, and the decline of the Ce^4+^ 3d_3/2_ signal (peak at ∼909 eV) at 25 and 127 °C under the MDR reaction condition indicates a slight reduction of Ce^4+^. This phenomenon was not observed on other similar 0.15 ML M/CeO_2_(111) (M = Co, Ni, and Cu) catalysts,^19^ implying a much stronger metal–support interaction in the Pt/CeO_2_(111) system. In addition, the slight reduction observed on the ceria support at 25 °C also manifests the increased reducibility of ceria when Pt was loaded, as there is no sign for CH_4_ dissociation on pure CeO_2_ at 25 °C as shown in Figure 2. This increased reducibility was also verified on the Pt/CeO_2_ powder system, as can be seen in Figure S1; the loading of Pt on the ceria support significantly decreased the temperature needed to reduce the surface of ceria.

As seen in Figure 6, as the temperature increases, a total shift of the Pt signal to lower binding energy at 327 and 427 °C was observed in the AP-XPS spectra, indicating the reduction of Pt^δ+^, and under the active MDR reaction conditions (> 400 °C), metallic Pt is present in the surface of the catalyst.^40,41^ The Ce^4+^ peak (at ∼909 eV) also grows at 227 °C and the signal of Ce^3+^ is negligible until 427 °C under the MDR reaction condition.^45^ In test experiments, we found that plain methane reduces ceria in the Pt/CeO_2_(111) system at elevated temperatures (∼23% of Ce^4+^ was reduced to Ce^3+^ at 427 °C, see Figure S2 and Table S1), but the formed Ce^3+^ quickly reoxidized to Ce^4+^ upon exposure to CO_2_. This indicates that a balanced redox cycle was achieved on the ceria support when the sample was exposed to the MDR reaction atmosphere. In general, under the active MDR reaction conditions, an interface containing small particles of Pt dispersed on a reactive ceria support is the active phase of the MDR catalyst.

### Reaction of CH_4_ and CO_2_ on Pt/CeO_2_ Powder

**Figure 7:**
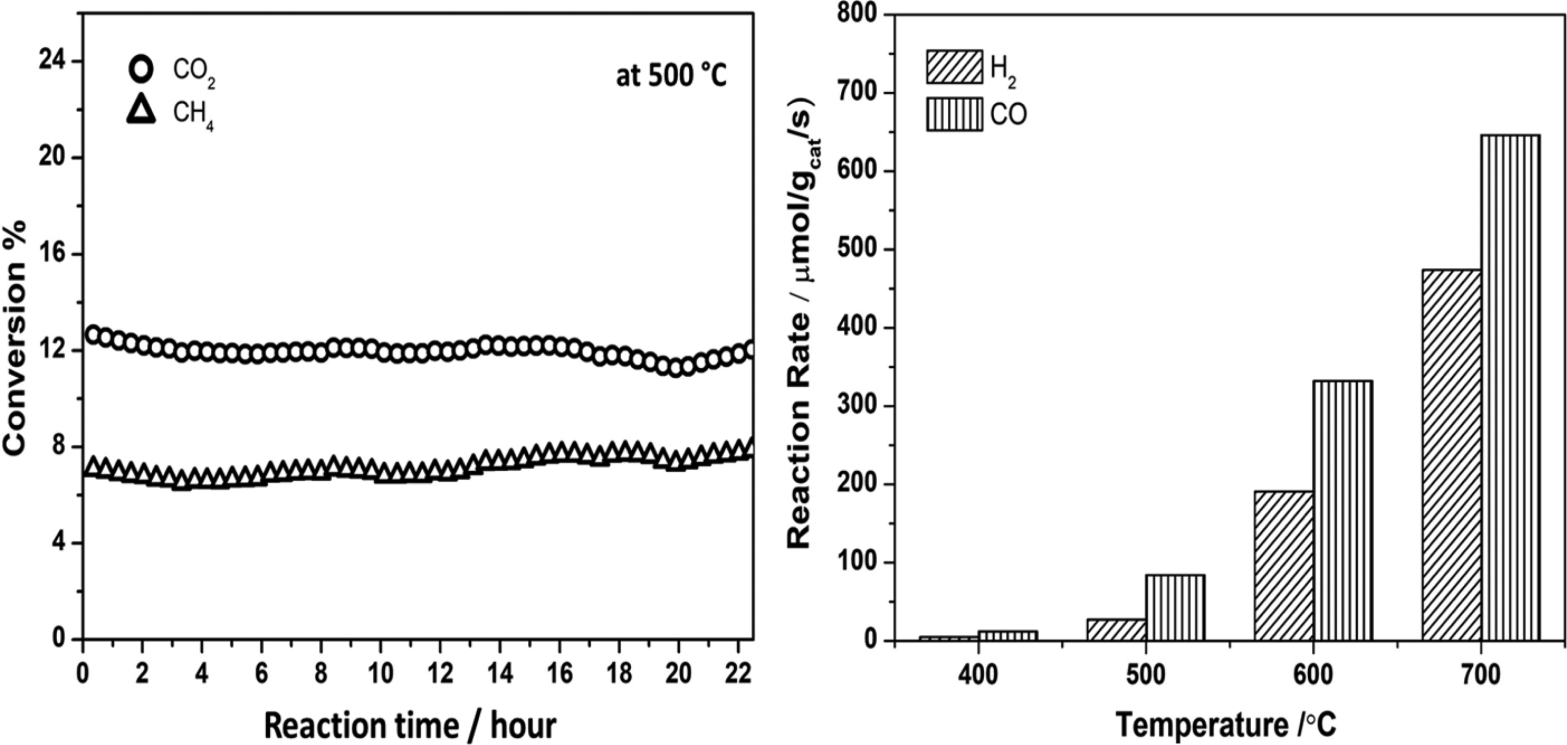
Catalytic performance of the 0.5 wt % Pt/CeO_2_ catalyst for the MDR reaction at different temperatures (400–700 °C).

Previous studies of AP-XPS and time-resolved X-ray diffraction (XRD) have shown that Pt/CeO_2_ powder is also effective for the low temperature activation of methane.^42^ Thus, we decided to test such a system in the conversion of CO_2_/CH_4_ and investigate the possible metal–support interactions in the Pt/CeO_2_ powder system under the MDR reaction condition. Tests in a flow reactor showed that a 0.5 wt % Pt/CeO_2_ powder was active and stable as an MDR catalyst at 500 °C with a very good performance at even higher temperatures (Figure 7). In the tests shown in Figure 7, CO_2_ was consumed by the MDR and reverse water-gas shift (CO_2_ + H_2_ → CO + H_2_O) reactions. At 500 °C, there was a 7% conversion of CH_4_ and 13% conversion of CO_2_, with the system remaining stable for more than 20 h. The production rate of H_2_ and CO was 27 and 84 μmol/g_cat_/s, respectively, at 500 °C and reached to 475 and 650 μmol/g_cat_/s at 700 °C.

**Figure 8:**
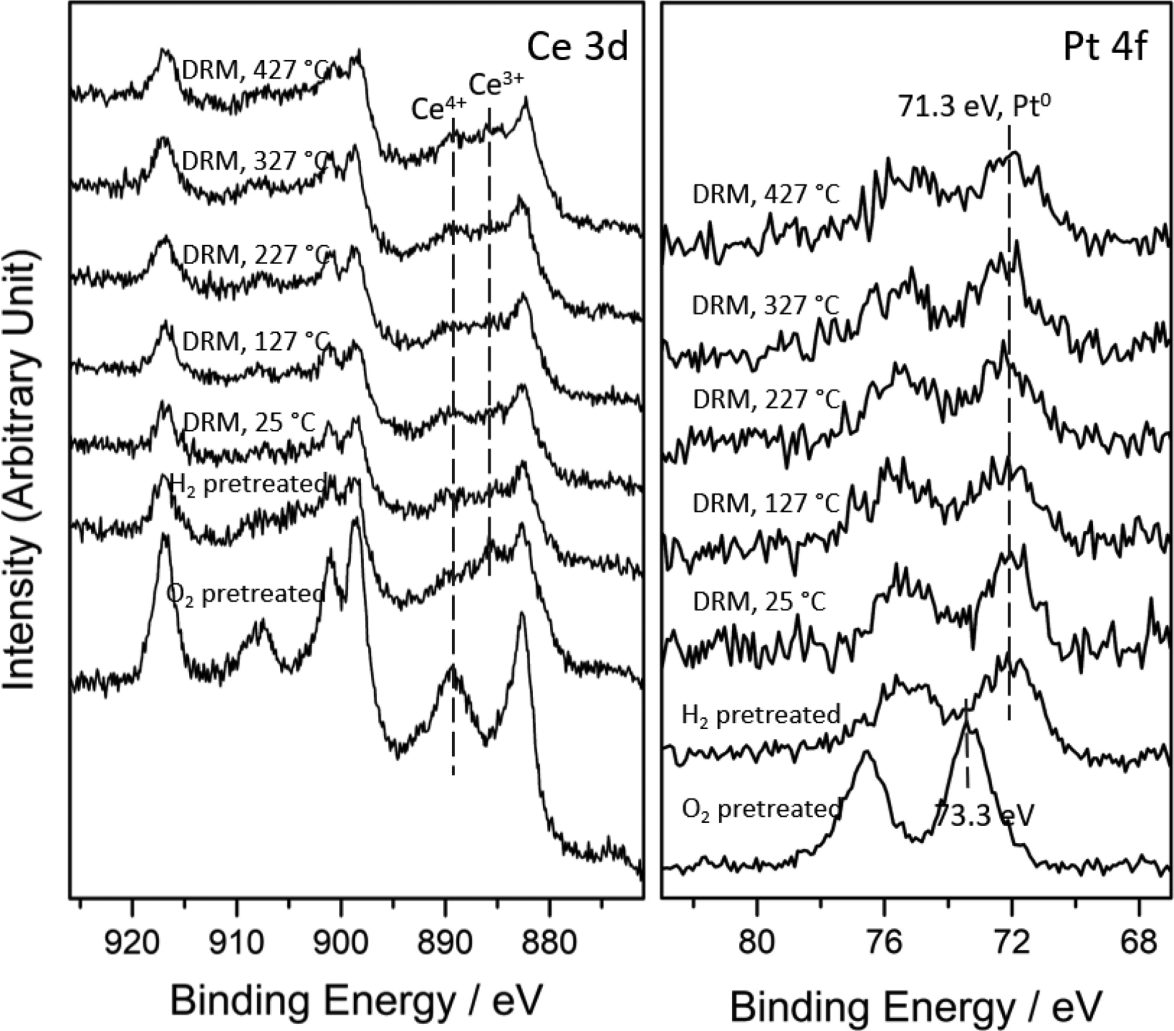
Ce 3d and Pt 4f AP-XPS spectra of 0.5 wt % Pt/CeO_2_ for the MDR reaction at elevated temperatures. A 10 mTorr O_2_ and a 20 mTorr of H_2_ were used to pretreat the sample at 400 °C sequentially to remove the surface-bounded carbon species and reduce the sample, respectively.

A combination of in situ measurements with AP-XPS, X-ray absorption fine structure (XAFS), and XRD was used to fully characterize the 0.5 wt % Pt/CeO_2_ powder catalyst under reaction. The AP-XPS results are summarized in Figure 8. Any Pt^δ+^ feature present on the samples at 25 °C was prereduced in H_2_ so that active metallic Pt was present on the catalysts surface for the MDR process. The prereduced Pt^0^ maintains its metallic feature throughout the MDR reaction at elevated temperatures. In the Ce 3d XPS region, the pretreatment process induced a partial reduction of Ce^4+^ to Ce^3+^; however, after switching gas to the MDR reaction gas mixture at 25 °C, part of Ce^3+^ was reoxidized back to Ce^4+^ by CO_2_, and under the reaction conditions, Ce^4+^ and Ce^3+^ kept a relatively stable ratio at elevated temperatures. On the surface of this catalyst, probably a dynamic redox process occurred under the MDR reaction conditions, where methane or H produced by methane dissociation reduced some Ce^4+^ to Ce^3+^, which was then partially reoxidized back by the dissociation of CO_2_.

**Figure 9:**
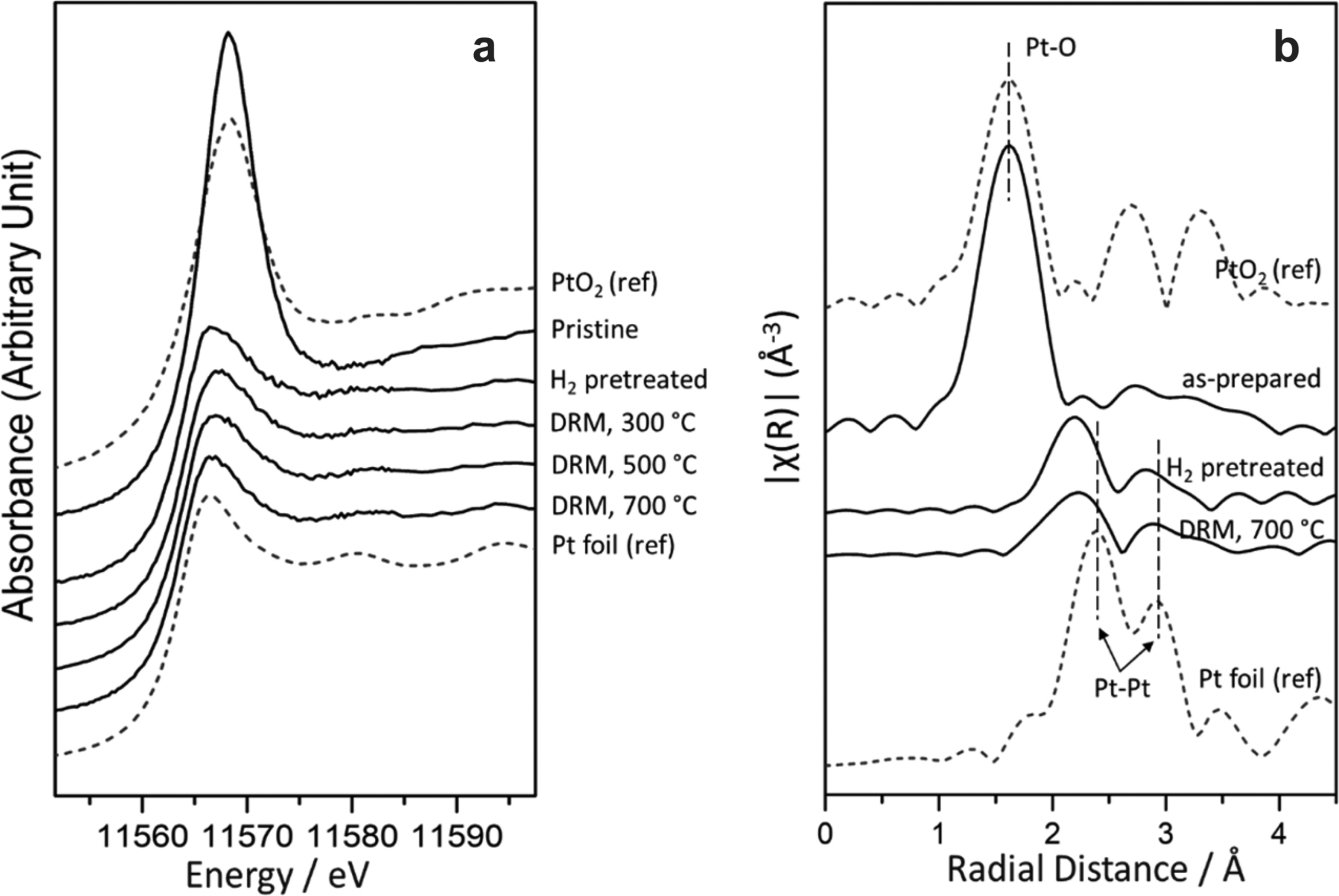
Pt L_3_ in situ XANES (a) and the Fourier transformed EXAFS region (b) of the sample during the MDR reaction at different temperatures. For comparison, we also include data for Pt foil and PtO_2_ powder.

A reduction of the supported platinum was also observed in X-ray absorption near edge structure (XANES) and extend X-ray absorption fine structure (EXAFS) measurements collected for the powder Pt/CeO_2_ catalyst using a flow reactor and the regular conditions for the MDR process. The Pt L_3_ edge XANES and the Fourier transformed EXAFS spectra are presented in Figure 9. PtO_2_ (Pt^4+^) was identified as a dominant structure in the as-prepared Pt/CeO_2_ powder sample,^46−48^ see Figure 9a. The strong Pt-O feature in the EXAFS spectrum of the pristine sample which aligns with the Pt-O characteristic peak of the PtO_2_ reference also confirms the initial presence of PtO_2_ in the bulk.^49^ After H_2_ reduction, PtO_2_ was converted to metallic Pt, which remained until 700 °C under the MDR reaction. In Figure 9b, a peak shift of ∼0.19 Å in the Pt-Pt shell was observed for the H_2_ pretreated sample and the sample under MDR conditions. This leftward shift implies shorter Pt–Pt bond distance of the small Pt clusters when they are supported on ceria, which evidenced the modification of Pt by the ceria support through the metal–support interactions in the powder Pt/CeO_2_ system.

**Figure 10:**
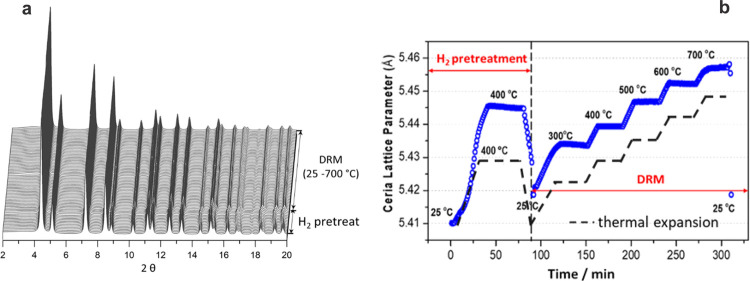
(a) Time-resolved in situ XRD profile of Pt/CeO_2_ and (b) evolution of the ceria lattice parameter during the MDR reaction.

Figure 10 shows in situ XRD data for 0.5 wt % Pt/CeO_2_ under the MDR process. Only diffraction features for ceria are observed in Figure 10a because the Pt particles are too small (< 2 nm) to yield diffraction lines. Rietveld refinement allowed us to track variations in the ceria lattice (Figure 10b). The XRD measurements point to an expansion of the ceria lattice indicating a reduction of the ceria support, which is also consistent with the existence of some Ce^3+^ in the powder catalyst as seen in AP-XPS (Figure 8). The ceria support was partially reduced by H_2_ with a 0.02 Å lattice expansion (from 5.41 to 5.43 Å) during the H_2_ pretreatment process. After switching the feed from H_2_ to an MDR reaction gas mixture at 25 °C, the ceria lattice decreased to 5.42 Å, indicating the partial reoxidation of the ceria support by CO_2_ at room temperature. Under the MDR reaction conditions, the ceria lattice expanded at elevated temperatures, following the thermal expansion trend of ceria, and after cooling down the sample to 25 °C, the ceria lattice contracted back to the same value as before the MDR reaction. When compared to the total lattice expansion of ceria under a pure CH_4_ gas environment from 25 to 700 °C (0.11 Å, in additional test measurements, shown in Figure S3), this result suggests that the partial reduction of the ceria support preserved a stable Ce^3+^ to Ce^4+^ ratio under the MDR reaction conditions (consistent with the results shown in Figure 8), and this implied that a balanced redox process, induced by simultaneous CH_4_ and CO_2_ decomposition, was achieved on the ceria support. This balanced redox process, also observed on the PtCeO_2_(111) model catalyst, is essential for the catalytic reaction. Although both reactants adsorb on the catalyst surface at 25 °C (Figures 2 and 3), a stable catalytic cycle is only established at elevated temperatures when methane or H produced by the dissociation of methane is able to reduce the ceria-forming Ce^3+^ sites which are effective for the dissociation of CO_2_.

### Comparison to Other Metal/Oxide Catalysts for the MDR Process

Small particles of Pt in contact with ceria display special electronic properties (valence photoemission data, Figure 1) and shorter Pt–Pt distances (EXAFS data, Figure 9) with respect to bulk Pt. These results are consistent with findings of previous studies examining the interaction of Pt atoms or small metal clusters with ceria.^35−37,45^ The electronic and structural perturbations affect the reactivity of the supported Pt particles. The results discussed above illustrate the cooperative interactions which can occur when a metal-oxide interface is used for the activation of CH_4_ and CO_2_ in a dry reforming process.

**Figure 11:**
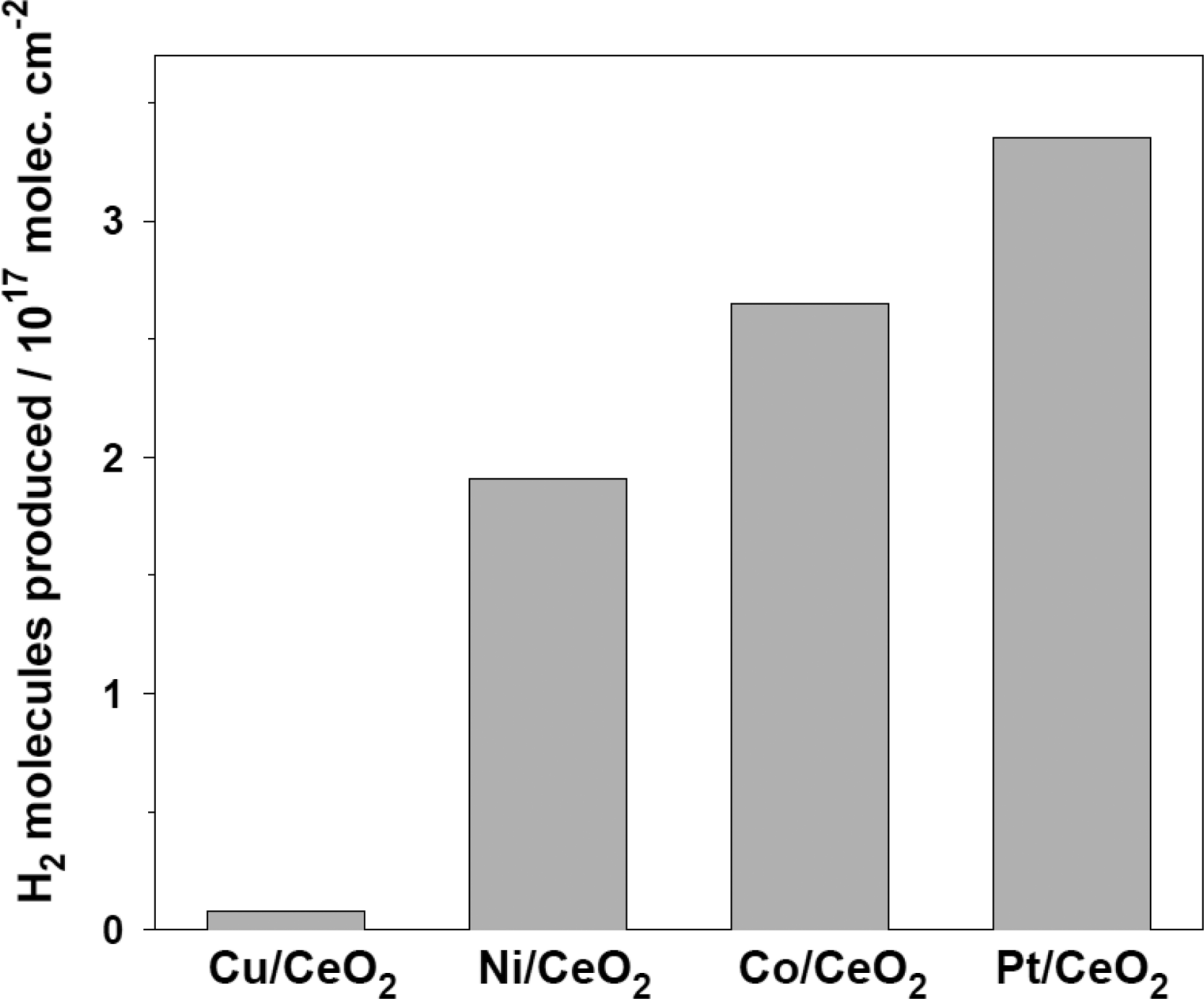
Catalytic activity for MDR on Cu-, Ni-, Co-, and Pt/CeO_2_ catalysts (∼0.15 ML of admetal). Amounts of H_2_ formed after exposing the catalysts to 1 Torr of CH_4_ and 1 Torr of CO_2_ at 650 K for 5 min.

In general, surfaces of pure platinum are not efficient for the activation of methane or carbon dioxide.^7,32−34^ The metal component alone cannot carry out the chemistry, and the oxide modifies the catalytic properties of the metal and participates in key reaction steps. In principle, the catalytic properties of the metal-oxide interface can be altered by changing its metal or oxide components. Figure 11 compares the catalytic activity of Pt/CeO_2_(111) and a series of M/CeO_2_(111) surfaces (M = Cu, Ni, or Co) at an admetal coverage of ∼0.15 ML.^18−20^ The high activity of Ni/CeO_2_ is remarkable;^50−52^ however, the surfaces with Co and Pt are clearly the best catalysts, with Pt being more selective than Co since it does not form ethane or ethylene during the MDR process, as it is the case with Co.^19^ Thus, in Pt/CeO_2_, one has the best metal-ceria system with high activity, stability, and selectivity.

In general, when optimizing the performance Pt MDR catalysts, scientists have followed two different approaches: changing the oxide support (Al_2_O_3_, MgO, CeO_2_, ZrO_2_, and CeO_2_-ZrO_2_) and alloying Pt with a second metal (Fe, Co, Ni, and Cu).^6,25−29,53−55^ Our results show the important role that ceria plays as an active support/participant in the MDR process. It is superior with respect to Al_2_O_3_ and MgO because these oxides usually do not interact strongly with supported metals like ceria does. Pure ZrO_2_ does interact with metals and produces an active ZrO_2_/Pt interface for the MDR reaction,^53^ but it does not have the redox properties of ceria which facilitate the dissociation of CO_2_ and its incorporation within a catalytic cycle. On the other hand, zirconia-doped ceria is an interesting support because it interacts well with metals and its redox properties (Ce^4+^ to Ce^3+^ switching) could be enhanced with respect to plain ceria; the increased stability against filamentous coke formation has also been reported on a Ni-based catalyst.^6,25,27,54,55^

Typically, surfaces of pure platinum {Pt(111), Pt(100), or Pt(110)-(1×2)} interact poorly with methane and, at high temperatures in which the breaking of C–H bonds is efficient, they become rapidly covered by a carbonaceous layer which leads to chemical and catalytic deactivation.^31−34^ The formation of metal–metal interfaces, or alloys, is an approach which is frequently used to prevent coke generation and subsequent deactivation during the MDR process.^6,8^ Pt is usually alloyed with a second metal (Ni, Co, Rh, Ru, or Ir) to increase the activity of the system for CO_2_ dissociation or to prevent carbon deposition and catalyst deactivation.^6,25,26,28,29^ In our Pt/CeO_2_ system, only a very small amount of Pt (0.15 ML and 0.5 wt %) was needed, and the chemical performance of the metal was promoted by interactions with the active oxide support, with ceria also helping in the dissociation of CO_2_. The results shown in Figures 2 and 3 are really remarkable because the common alloys of Pt investigated so far have not been able to bind CO_2_ and CH_4_ well or activate them in an effective way at room temperature.^6,25−29,56,57^ The TOFs seen in Figure 5 (17–19 H_2_ molecules produced site^–1^ s^–1^) are much larger than typical TOFs obtained after alloying Pt with a second metal (2–5 H_2_ molecules produced site^–1^ s^–1^).^29,58,59^ Thus, optimizing metal–support interactions and using an active oxide support seem a much more efficient approach than plain bimetallic bonding when one wants to produce a highly active, selective, and stable catalyst for the MDR process.

## Conclusions

Pt(111) reacts poorly with carbon dioxide and methane. Elevated temperatures are necessary to activate these molecules, and a massive deposition of carbon made this pure metal surface useless for the MDR process. The deposition of small Pt particles on ceria produces systems with short Pt–Pt distances and induces large electronic perturbations in the valence states of the admetal, evidencing strong metal–support interactions in Pt/CeO_2_(111) and Pt/CeO_2_ powders, leading to systems which bind CO_2_ and CH_4_ well at room temperature and are excellent and stable catalysts for the MDR process at moderate temperatures (500 °C). Studies with in situ or *operando* methods (AP-XPS, XRD, and XAFS) point to an active Pt-CeO_2-*x*_ interface. In this interface, the oxide is not only a passive spectator but also modifies the chemical properties of Pt, facilitating methane dissociation, and is directly involved in the adsorbing and dissociation of CO_2_, making the MDR catalytic cycle possible. A comparison of the benefits gained by the use of an effective metal-oxide interface and those obtained by plain bimetallic bonding indicates that the former is much more important when optimizing the C1 chemistry associated with CH_4_ and CO_2_ conversion. The presence of elements with a different chemical nature at the metal-oxide interface opens up the possibility for truly cooperative interactions in the activation of C–H and C–O bonds.

## Methods

### Studies with Well-Defined Pt/CeO_2_(111) Surfaces

The experiments examining the activation of CH_4_ and its conversion by reaction with CO_2_ on Pt/CeO_2_(111) surfaces were performed in a setup that combined an ultrahigh vacuum (UHV) chamber for surface characterization and a microreactor for catalytic tests.^18,19,37^ The UHV chamber was equipped with instrumentation for XPS, low-energy electron diffraction, ion-scattering spectroscopy, and thermal-desorption mass spectroscopy.^18,19,37^ The methodology followed for the preparation of the Pt/CeO_2_(111) surfaces is described in detail in ref.^37^ For Pt/CeO_2_(111) surfaces, single atoms and small Pt clusters have been observed at low coverages using scanning tunneling microscopy.^60^ In the studies of methane activation, the sample was transferred in vacuo to the reactor at 25 °C and then the reactant gas, 1 Torr of pressure, was introduced. In the experiments of testing the activity of Pt(111) and Pt/CeO_2_(111) catalysts for the MDR reaction, the samples were under a gas mixture of 1 Torr of CH_4_ and 1 Torr of CO_2_ at room temperature and were heated in a fast ramp to a reaction temperature of 500 °C. The MDR products were analyzed by mass spectroscopy or gas chromatography. In our catalytic experiments, yields were measured at intervals of 5 min. For each run, the number of molecules (CO and/or H_2_) generated in the kinetic tests was normalized by the active area exposed by the sample and the total reaction time. All these kinetic experiments in a batch reactor were done under a limit of low conversion (< 10%).

AP-XPS measurements were carried out on a commercial SPECS AP-XPS chamber equipped with a PHOIBOS 150 EP MCD-9 analyzer at the Chemistry Division of Brookhaven National Laboratory (BNL). In the preparation of the Pt/CeO_2_(111) model catalyst, the Ce metal was first evaporated onto a Ru single crystal (0001) at 427 °C in the presence of 5 × 10^–7^ torr O_2_ and then annealed to 527 °C for 10 mins at the same O_2_ pressure. The ceria films were estimated to be ca. 4 nm thick (≈10 layers of O-Ce-O) based on the attenuation of the Ru 3d XPS signal. Pt was vapor-deposited on the as-prepared ceria film at 427 °C, and the coverage of Pt was ∼0.15 ML, estimated by the attenuation of the Ce 3d XPS signal. In the studies of MDR on the Pt/CeO_2_(111) catalyst, a 50 mTorr CH_4_ and 50 mTorr CO_2_ gas mixture was used and Ce 3d and Pt 4f spectra were collected at 25, 127, 227, 327, and 427 °C. The binding energies in the AP-XPS spectra were calibrated using the strongest Ce^4+^ 3d feature located at 916.9 eV as a reference.

### Studies with Pt/CeO_2_ Powder Catalysts

#### Catalyst Preparation

Cerium oxide (CeO_2_) was prepared by precipitating white crystalline cerous nitrate (Ce(NO_3_)_3_.6H_2_O; Sigma-Aldrich), dissolved in deionized water with mild stirring. The temperature was kept at 100 °C and ammonia (0.91 molL^–1^) was added dropwise until a pH of 8 was attained. The resulting white slurry precipitate was then collected by filtration, washed with deionized water, and left to dry in an oven at 100 °C for 12 h. The pale purple dried powder was calcined in a furnace at 500 °C for 4 h with flowing air. The sample was then ground but not sieved to a consistent powder with a mortar and pestle. The required amount of Pt to make 0.5 wt % Pt/CeO_2_ (0.05 g of Pt on 9.95 g of CeO_2_) was deposited on CeO_2_ by impregnation from a stock solution of 1 L/g of Pt cations (from PtCl_4_—Sigma-Aldrich) in a beaker at ambient temperature with continuous stirring. The temperature of the beaker was then raised to around 100 °C, while stirring, and retained at this temperature until most of the liquid has vaporized (the complete impregnation process takes about 6–8 h) to form a pastelike material, which was then dried in an oven for 12 h at 100 °C to remove the remaining water. The dried powder was then calcined in a furnace for 4 h at 400 °C under flowing air.

#### Catalytic Performance

In the catalytic test for the (0.5 wt %) Pt/CeO_2_ powder catalyst under the MDR reaction, a sample of ∼12.5 mg was loaded into a quartz tube flow reactor for the measurements. The catalysts were pretreated with H_2_ at 400 °C for 40 min to generate active metallic Pt and then an MDR reaction gas mixture (10 cc/min CH_4_, 10 cc/min CO_2_, and 10 cc/min N_2_) was switched into the flow system for the reaction test. The weight hourly space velocity was 180,000 mL/g_cat_/h. The catalysts were heated to 700 °C with a 10 °C/min ramping rate and isothermal stages at 400, 500, 600, and 700 °C, and at each temperature stage, the soak time was 1 h. A residual gas analyzer and a gas chromatography instrument (Agilent 7890A) were connected to the end of the flow reactor to analyze the reaction gas products and the catalytic activity was measured and calculated at each of the isothermal stages (400, 500, 600, and 700 °C) investigated.

#### XAFS

In situ XANES and EXAFS for the MDR reaction on Pt/CeO_2_ were recorded at 9BM of the Advanced Photon Source (APS), at Argonne National Laboratory (ANL). Around 2 mg of the catalysts was loaded into a Clausen cell flow reactor and directly placed in front of the synchrotron X-rays for the in situ measurement. The catalysts were pretreated in H_2_ at 400 °C for 40 min before switching to a 2 cc/min CH_4_, 2 cc/min CO_2_, and 6 cc/min He gas mixture for the MDR reaction testing. The catalysts were then heated to 700 °C with a 10 °C/min ramping rate and the Pt L_3_-edge spectra were collected in the fluorescence yield mode at temperature stages of 300, 500, and 700 °C by a four channel Vortex detector. At least three spectra at each temperature stage were collected and averaged to improve the data quality, and these spectra were further processed using the IFEFFIT package.^61^

#### AP-XPS

The powder catalyst was pressed onto an aluminum plate and loaded on a sample holder in the AP-XPS chamber. A 10 mTorr of O_2_ was introduced and the sample was heated to 400 °C to remove any surface-bounded carbon species before the test. In the studies of MDR on the Pt/CeO_2_ powder catalyst, the sample was prereduced in a 20 mTorr of H_2_ at 400 °C for 40 min before switching to a 50 mTorr CH_4_ and 50 mTorr CO_2_ reaction gas mixture. The Pt 4f and Ce 3d XPS signals were collected at 25, 127, 227, 327, and 427 °C.

#### XRD

A Clausen cell flow reactor was used for the in situ time-resolved XRD studies.^62^ The measurements were conducted at the 17BM beamline of the Advanced Photon Source (APS), at Argonne National Laboratory (ANL), with an X-ray wavelength at 0.24108 Å. The reaction conditions were kept the same as those for the in situ XAFS measurements. Two-dimensional XRD images were continuously collected by an amorphous Si flat panel (PerkinElmer) detector throughout the reaction process and the XRD images were further processed with the GSAS-II code to generate XRD patterns (Intensity versus 2θ). The lattice parameter evolution of ceria was calculated by Rietveld refinement also using GSAS-II.^63^ Pt particles or aggregates were not seen in XRD and TEM for the 0.5 wt % Pt/CeO_2_ powder catalyst. The use of low loading is crucial for the comparison of model systems with high surface area catalysts and for defining structure–function relationships.^64^

## Supporting Information Available

The H_2_ TPR results on the Pt/CeO_2_ powder sample and bare ceria support; AP-XPS signal and the peak fitting at the Ce 3d region of 0.15 ML Pt/CeO_2_(111) under a pure CH_4_ atmosphere at 427 °C; fitting parameters and results of the Ce 3d spectrum; ceria lattice parameter evolution in a CH_4_ atmosphere as a function of temperature (PDF)

## Notes

The data that support the plots presented in this paper and other findings of this study are available from the corresponding authors on reasonable request.
