# Supplementary material for: Metal–Support Interactions and C1 Chemistry: Transforming Pt-CeO2 into a Highly Active and Stable Catalyst for the Conversion of Carbon Dioxide and Methane
Source: ACS Catal. 2021 Jan 20;11(3):1613–23. doi: 10.1021/acscatal.0c04694 (PMC8210818; doi:10.1021/acscatal.0c04694)
Supplement: Supplementary file 1 — cs0c04694_si_001.pdf [file cs0c04694_si_001.pdf]

Supporting Information

Metal-support Interactions and C1 Chemistry: Transforming Pt-CeO_2 into a Highly Active and Stable Catalyst for the Conversion of Carbon Dioxide and Methane

Feng Zhang,[a] Ramón A. Gutiérrez,[b] Pablo G. Lustemberg,[c,d] Zongyuan Liu,[e] Ning Rui,[e] Tianpin Wu,[f] Pedro J. Ramírez,[b,g] Wenqian Xu,[f] Hicham Idriss,[h] M. Verónica Ganduglia-Pirovano,*[d] Sanjaya D. Senanayake,*[e] and José A. Rodriguez*[a,e]

[a] Department of Materials Science and Chemical Engineering, SUNY at Stony Brook, Stony Brook, NY 11794 (United States)

[b] Facultad de Ciencias, Universidad Central de Venezuela, Caracas 1020-A (Venezuela)

[c] Instituto de Fisica Rosario (IFIR), CONICET-UNR, Bv. 27 de Febrero 210bis, S2000EZP Rosario, Santa Fe, (Argentina)

[d] Instituto de Catálisis y Petroleoquímica, CSIC, C/Marie Curie 2, 28049 Madrid (Spain)

[e] Chemistry Division, Brookhaven National Laboratory, Upton, NY 11973 (United States)

[f] X-ray Science Division, Advanced Photon Source, Argonne National Laboratory, Argonne, Illinois 60439 (United States)

[g] Zoneca-CENEX, R&D Laboratories, Alta Vista, 64770 Monterrey (México)

[h] SABIC Corporate Research & Development (CRD), KAUST, Thuwal, 29355 (Saudi Arabia)

*Corresponding authors: M. Verónica Ganduglia-Pirovano (vgp@icp.csic.es); Sanjaya D. Senanayake (ssenanay@bnl.gov); José A. Rodriguez (rodrigez@bnl.gov)

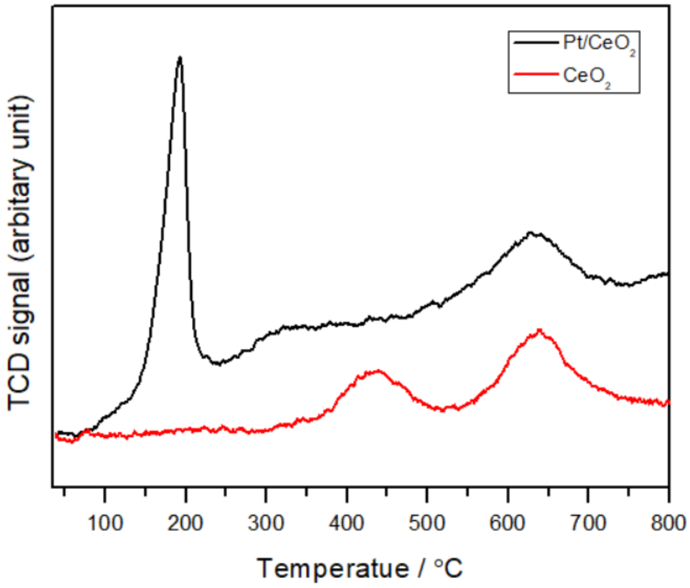

Figure S1. The H_2 TPR results on Pt/CeO_2 powder sample and bare ceria support.

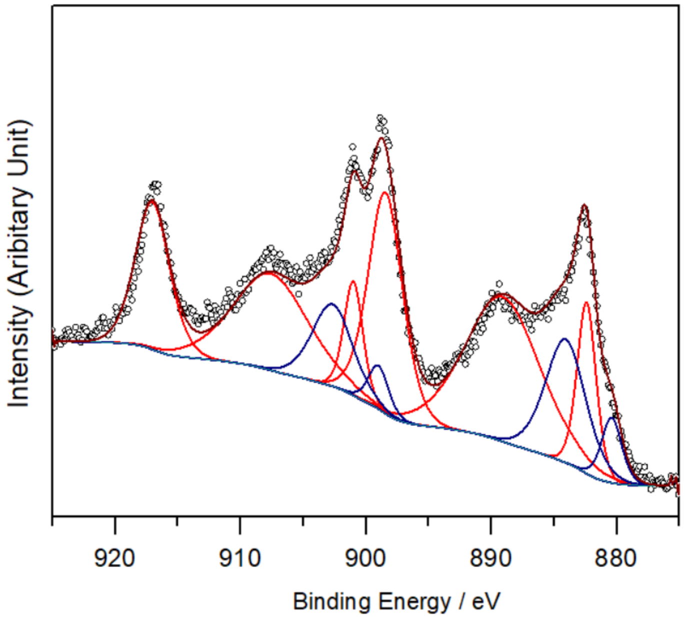

Figure S2. AP-XPS signal and the peak fitting at Ce 3d region of 0.15 ML Pt/CeO_2(111) under a pure CH_4 atmosphere at 427 °C. The red and blue lines in figure indicates Ce^{4+} and Ce^{3+}, respectively.

Table S1. Fitting parameters and results of the Ce 3d spectrum in Figure S2.

Component	Position	FWHM	Area	%Area	%4+ & 3+	%StDev
Ce 4+	882.41	1.78	7619.76	6.96		2.04
Ce 4+	901.01	1.78	5082.38	4.64		1.36
Ce 4+	889.1	7	25387.27	23.20	77.47	1.23
Ce 4+	907.6	7	16933.31	15.48		0.82
Ce 4+	898.44	3.22	17846.34	16.31		0.82
Ce 4+	917.04	3.22	11903.51	10.88		0.55
Ce 3+	880.41	1.91	3101.61	2.83		0.84
Ce 3+	899.01	1.91	2068.78	1.89	22.53	0.56
Ce 3+	884.07	3.79	11684.83	10.68		2.46
Ce 3+	902.67	3.79	7793.78	7.12		1.64
Total Residual STD is 1.20%						

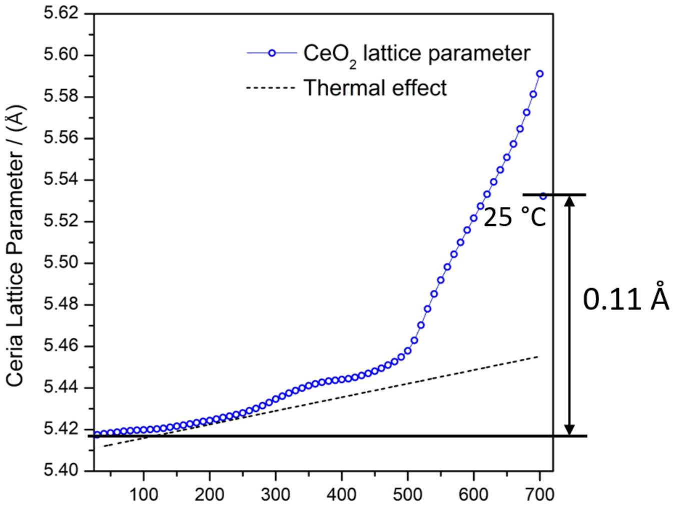

Figure S3. Ceria lattice parameter evolution in a CH_4 atmosphere as a function of temperature.
